# Supplementary material for: Association between Glycosylated Hemoglobin Levels and Vaccine Preventable Diseases: A Systematic Review
Source: Diseases. 2024 Aug 17;12(8):187. doi: 10.3390/diseases12080187 (PMC11353432; doi:10.3390/diseases12080187)
Supplement: Supplementary file 1 [file diseases-12-00187-s001.zip › diseases-2997701-supplementary.pdf]

Supplementary Table S1. Search strategies.

Database (number)	Search strategy
**Pubmed (372)**	(“Glycated Hemoglobin” [tiab] OR Glycohemoglobin [tiab] OR “Glycosylated Hemoglobin” [tiab] OR Hb A1c [tiab] OR HbA1c [tiab]) AND (Measles [tiab] OR morbilli* [tiab] OR Mumps [tiab] OR Parotitis [tiab] OR Rubella [tiab] OR Polio [tiab] OR Poliomyelitis [tiab] OR Diphtheria [tiab] OR “Corynebacterium diphtheriae” [tiab] OR Tetanus [tiab] OR “Clostridium tetani” [tiab] OR Pertussis [tiab] OR “Whooping Cough” [tiab] OR Hepatit* [tiab] OR “Haemophilus influenzae” [tiab] OR “Influenza-bacillus” [tiab] OR “Hemophilus influenzae” [tiab] OR “Pneumococcal Disease” [tiab] OR Pneumococcus [tiab] OR “Streptococcus pneumoniae” [tiab] OR Influenza [tiab] OR Flu [tiab] OR Varicella [tiab] OR “Herpes Zoster” [tiab] OR Shingles [tiab] OR “Human Papillomavirus” [tiab] OR HPV [tiab] OR “Meningococcal Disease” [tiab] OR Meningococcus [tiab] OR “Neisseria meningitidis” [tiab] OR monkeypox [tiab] OR “monkey pox” [tiab])
**Embase (501)**	('measles'/exp OR 'measles' OR 'morbilli' OR 'rubeola' OR 'parotitis'/exp OR 'chronic parotitis' OR 'chronic recurrent parotitis' OR 'parotid inflammation' OR 'parotitis' OR 'rubella'/exp OR 'epidemic roseola' OR 'german measles' OR 'infection of rubella' OR 'measles, german' OR 'rubella' OR 'rubella infection' OR 'rubella virus infection' OR 'tropical rubella' OR 'poliomyelitis'/exp OR 'heine-medin disease' OR 'heine-medin`s disease' OR 'acute anterior poliomyelitis' OR 'anterior acute poliomyelitis' OR 'polio' OR 'polio virus infection' OR 'polio virus infections' OR 'polio-myelitis' OR 'poliomyelites' OR 'poliomyelitis' OR 'poliomyelitis anterior acuta' OR 'poliovirus infection' OR 'poliovirus infections' OR 'diphtheria'/exp OR 'c. diphtheriae infection' OR 'corynebacterium diphtheriae infection' OR 'diphteria' OR 'diphtheria' OR 'infection by corynebacterium diphtheriae' OR 'infection caused by corynebacterium diphtheriae' OR 'infection due to corynebacterium diphtheriae' OR 'tetanus'/exp OR 'c. tetani infection' OR 'clostridium tetani infection' OR 'clostridial tetanus' OR 'infection by clostridium tetani' OR 'infection caused by clostridium tetani' OR 'infection due to clostridium tetani' OR 'tetanus' OR 'pertussis'/exp OR 'b. pertussis disease' OR 'b. pertussis infection' OR 'bordetella pertussis disease' OR 'bordetella pertussis infection' OR 'cough, whooping' OR 'infection by b. pertussis' OR 'infection by bordetella pertussis' OR 'infection caused by b. pertussis' OR 'infection caused by bordetella pertussis' OR 'pertussis' OR 'whooping cough' OR 'whooping cough syndrome' OR 'streptococcus pneumoniae'/exp OR 'diplococcus pneumoniae' OR 'micrococcus pneumoniae' OR 'pneumococcus' OR 'pneumococcus pneumococcus' OR 'pneumococcus pneumoniae' OR 'streptococcus pneumoniae' OR 'influenza'/exp OR 'bronchitis epidemica' OR 'bronchitis, epidemic' OR 'epidemic bronchitis' OR 'flu' OR 'flue' OR 'human influenza' OR 'infection caused by influenza virus' OR 'infection caused by influenzavirus' OR 'influenza' OR 'influenza infection' OR 'influenza syndrome' OR 'influenza virus infection' OR 'influenza, human' OR 'influenzavirus infection' OR 'chickenpox'/exp OR 'chicken pox' OR 'chickenpox' OR 'varicella' OR 'varicella infection' OR 'varicellas' OR 'neisseria meningitidis'/exp OR 'diplococcus intracellularis meningitidis' OR 'meningococcus' OR 'micrococcus intracellularis' OR 'micrococcus meningitidis' OR 'neisseria meningitides' OR 'neisseria meningitidis' OR 'neisseria meningitidis serogroup y' OR 'neisseria meningitidis, serogroup w-135' OR 'neisseria meningitidis, serogroup y' OR 'neisseria weichselbaumi' OR 'meningococci' OR 'serogroup w-135 neisseria meningitidis' OR 'serogroup y neisseria

Database (number)	Search strategy
	meningitidis') AND ('hemoglobin a1c'/exp OR 'hb a1c' OR 'glycated haemoglobin a1c' OR 'glycated hemoglobin a1c' OR 'glycosylated haemoglobin a1c' OR 'glycosylated hemoglobin a1c' OR 'haemoglobin a1c' OR 'haemoglobin a (1c)' OR 'haemoglobin a 1c' OR 'haemoglobin aic' OR 'hb a (1c)' OR 'hba 1c' OR 'hba1c' OR 'hemoglobin a1c' OR 'hemoglobin a (1c)' OR 'hemoglobin a 1c' OR 'hemoglobin aic')

Supplementary Table S2. Excluded references, with reason.

Article	Reason
Breitling LP (2016) Evidence of non-linearity in the association of glycemic control with influenza/pneumonia mortality: a study of 19 000 adults from the US general population. Diabetes Metab Res Rev 32:111-120	No outcome of interest
Cai T, Yue T, Xu M, et al. (2023) Poor glycemic control in type-2 diabetic patients infected with hepatitis B: A retrospective propensity-matched study. J Med Virol 95:e28635	No outcomes of interest
Carey RAB, Ch, iraseharan VK, Jasper A, Sebastian T, Gujjarlamudi C, Sathyendra S, Zachariah A, Abraham AM, Sudarsanam TD (2017) Varicella Zoster Virus Infection of the Central Nervous System - 10 Year Experience from a Tertiary Hospital in South India. Ann Indian Acad Neurol 20:149-152	No study design (case series)
Ebekozien OA, Noor N, Gallagher MP, Alonso GT (2020) Type 1 diabetes and covid-19: Preliminary findings from a multicenter surveillance study in the U.S. Diabetes Care 43:e83-e85	No outcomes of interest
Heald AH, Stedman M, Horne L, Rea R, Whyte M, Gibson JM, Livingston M, Anderson SG, Ollier W (2021) Analysis of Continuous Blood Glucose Data in People with Type 1 Diabetes (T1DM) After COVID-19 Vaccination Indicates a Possible Link Between the Immune and the Metabolic Response. Journal of Diabetes Science and Technology 15:1204-1205	No outcomes of interest
Hine JL, de Lusignan S, Burleigh D, et al. (2017) Association between glycaemic control and common infections in people with Type 2 diabetes: a cohort study. Diabet Med 34:551-557	No population and no outcome of interest
Ho FK, Celis-Morales CA, Gray SR, et al. (2020) Modifiable and non-modifiable risk factors for COVID-19, and comparison to risk factors for influenza and pneumonia: Results from a UK Biobank prospective cohort study. BMJ Open 10:	No outcome of interest
Hopkins R, Young KG, Godwin J, Raja D, Thomas NJ, Shields BM, Dennis JM, McGovern AP (2022) Modifiable risk factors including HbA1c and BMI are consistently associated with severe influenza, pneumonia, and Covid-19 infection outcomes in people with type 2 diabetes. Diabetologia 65:S123-S124	No study design
Hsiang JC, Gane EJ, Bai WW, Gerred SJ (2015) Type 2 diabetes: a risk factor for liver mortality and complications in hepatitis B cirrhosis patients. J Gastroenterol Hepatol 30:591-599	No outcome of interest
Katipoglu B, Naharci MI, Yurdakul ES (2022) Risk factors predicting hospital length of stay in older patients with type 2 diabetes with Covid-19. Journal of diabetes and metabolic disorders 21:1443-1449	No outcome of interest
Muñoz-Quiles C, López-Lacort M, Ampudia-Blasco FJ, Díez-Domingo J (2017) Risk and impact of herpes zoster on	No outcome of interest

Article	Reason
patients with diabetes: A population-based study, 2009-2014. Hum Vaccin Immunother 13:2606-2611	
Shimada S, Kamiyama T, Orimo T, Nagatsu A, Kamachi H, Taketomi A (2021) High HbA1c is a risk factor for complications after hepatectomy and influences for hepatocellular carcinoma without HBV and HCV infection. Hepatobiliary Surg Nutr 10:454-463	No population
Yang X, Wang Y, Luk AO, So WY, Ma RC, Kong AP, Xu G, Chan JC (2013) Enhancers and attenuators of risk associations of chronic hepatitis B virus infection with hepatocellular carcinoma in type 2 diabetes. Endocr Relat Cancer 20:161-171	No outcome of interest

Supplementary Table S3. Evaluation of the risk of bias.

NOS criteria	**Studies (year)**		
	Kobayashi, 2019	***De Jong, 2021***	***Pan,2022***
A. Selection (maximum of four stars)			
1. Representativeness of the exposed cohort			
2. Selection of the non-exposed cohort	★	★	★
3. Ascertainment of exposure	★	★	★
4. Demonstration that outcome of interest was not present at start of study (no VPDs at start of study)	★	★	★
B. Comparability (maximum of two stars)			
1. Comparability of cohort on the basis of the design or analysis	★★	★★	★★
C. Outcome (maximum of three stars)			
1. Assessment of outcome	★	★	★
2. Was follow-up long enough for outcomes to occur		★	★
3. Adequacy of follow-up of cohorts			
**Total (maximum of nine stars)**	**6**	**7**	**7**
